# Esophageal cancer treated by endoscopic submucosal dissection after neoadjuvant chemoradiotherapy: a case report

**DOI:** 10.1186/1752-1947-8-439

**Published:** 2014-12-18

**Authors:** Jang Hwan Lim, Sang Ah Lee, Gyoun Eun Kang, Jong Min Kim, Jin Kyung Park, Yun Jin Chung, Jae Kwon Jung, Chang Keun Park, Hyun Soo Kim, Dong Wook Lee

**Affiliations:** Division of Gastroenterology and Hepatology, Department of Internal Medicine, Daegu Fatima Hospital, 183 Ayangro, Dong-gu, Daegu, 701-600 South Korea

**Keywords:** Endoscopic submucosal dissection, Esophageal cancer, Neoadjuvant chemoradiotherapy

## Abstract

**Introduction:**

The treatment of esophageal cancer remains clinically challenging because of the overall poor prognosis associated with the disease. The mortality rate associated with surgical treatment is high, and the majority of diagnosed patients are old. As such, surgery is not possible in many cases, even when the cancer has progressed to a resectable state.

**Case presentation:**

We present the case of an 82-year-old Korean man who presented to our institution with intermittent odynophagia. Esophageal cancer with submucosal invasion and metastasis to three regional lymph nodes was diagnosed. After neoadjuvant chemoradiotherapy, his regional lymph nodes disappeared. Because of his poor pulmonary function, surgical treatment could not be performed. Endoscopic submucosal dissection was carried out instead, and endoscopic triamcinolone injections were performed serially. Neither recurrence nor abnormal symptoms such as dysphagia or regurgitation have developed for 36 months.

**Conclusions:**

The literature suggests that endoscopic submucosal dissection after chemoradiotherapy is a viable treatment modality in patients with esophageal cancer with a high surgical treatment risk.

## Introduction

Esophageal cancer is a highly lethal malignancy with a 5-year survival rate of approximately 15% [1]. Thus, the management of patients with esophageal cancer is challenging and requires a multimodal approach. Suboptimal results have been reported for post-operative adjuvant chemotherapy [2], radiotherapy [3] and concurrent chemoradiotherapy [4]; thus, interest in neoadjuvant treatment has recently increased. In particular, Sjoquist *et al*. reported that, among patients with resectable esophageal cancer, those who received neoadjuvant chemoradiotherapy showed a significant survival benefit compared to patients who received surgical treatment alone [5]. However, the incidence of esophageal cancer and squamous cell carcinoma is known to increase with age. The incidence of cancer peaks in the seventh decade of human life [6]. Thus, even if resection is possible, surgical treatment cannot be performed in some patients because of their general condition or accompanying comorbidities at the time of diagnosis. In this report, we describe the case of a patient with esophageal cancer who could not undergo surgical treatment because he also had chronic obstructive pulmonary disease (COPD). We successfully treated the patient with endoscopic submucosal dissection (ESD) after concurrent chemoradiotherapy. Our description of the case is accompanied by a discussion of relevant literature.

## Case presentation

An 82-year-old Korean man was admitted to our hospital with a chief complaint of intermittent odynophagia of approximately 3 months’ duration. The patient did not have a history of alcohol intake. He was a current smoker with a smoking history of 120 pack-years (2 packs daily for 60 years). He had been taking medication and using an inhaler for approximately 20 years to treat his COPD. A pulmonary function test (performed approximately 2 months prior to his presentation) revealed poor respiratory function with a forced expiratory volume in 1 second of approximately 45%.

**Figure 1:**
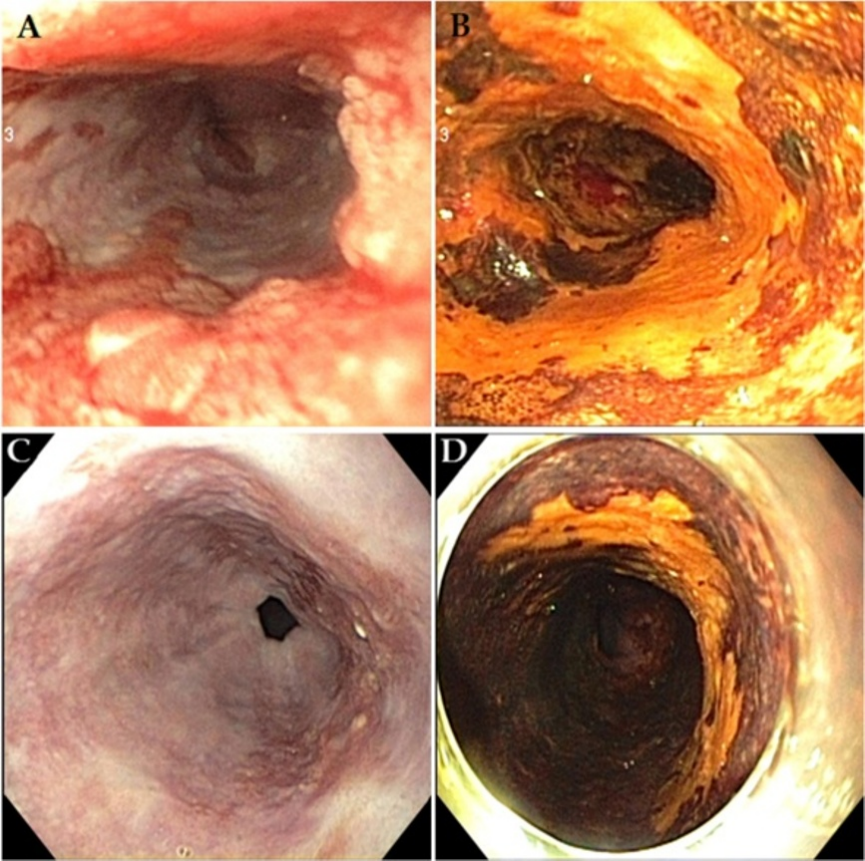
**Endoscopic examination.** Esophageal cancer was observed at the middle to distal esophagus. It spanned more than three-fourths of the luminal circumference. **(A)** White light endoscopy. **(B)** Chemoendoscopy with iodine staining. After chemoradiotherapy, the lesion size decreased to approximately half of the circumference of the esophageal lumen. **(C)** White light endoscopy. **(D)** Chemoendoscopy with iodine staining.

**Figure 2:**
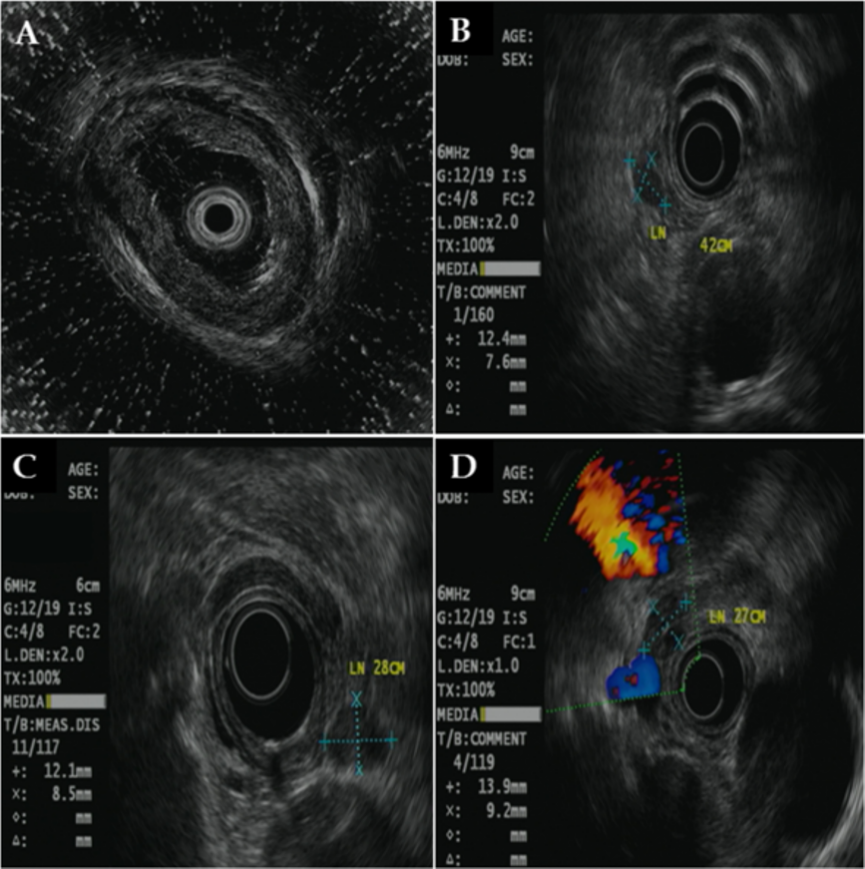
**Endoscopic ultrasonography findings. (A)** Blurring and thickening of the submucosal layer were observed, but the proper muscle layer was spared. Three enlarged regional lymph nodes (>10mm) were observed at 42cm **(B)**, 28cm **(C)** and 27cm **(D)** from the incisor.

An endoscopic examination showed a broad, ulcerative lesion at 33cm to 38cm from the incisor that spanned more than three-fourths of the esophageal lumen circumference (Figures 1A and 1B). The biopsy findings indicated moderately differentiated squamous cell carcinoma. Endoscopic ultrasonography (EUS) revealed a blurring and thickening of the third layer (submucosal layer). However, the fourth layer (proper muscle layer) was spared. In addition, we observed three malignant lymph nodes >10mm that were round in shape, had smooth features and exhibited hypoechogenicity. They were located 27cm, 28cm and 42cm from the incisor (Figures 2A, 2B, 2C and 2D). However, we did not find any metastases on a computed tomography (CT) scan or a positron emission tomography (PET)-CT scan.

**Figure 3:**
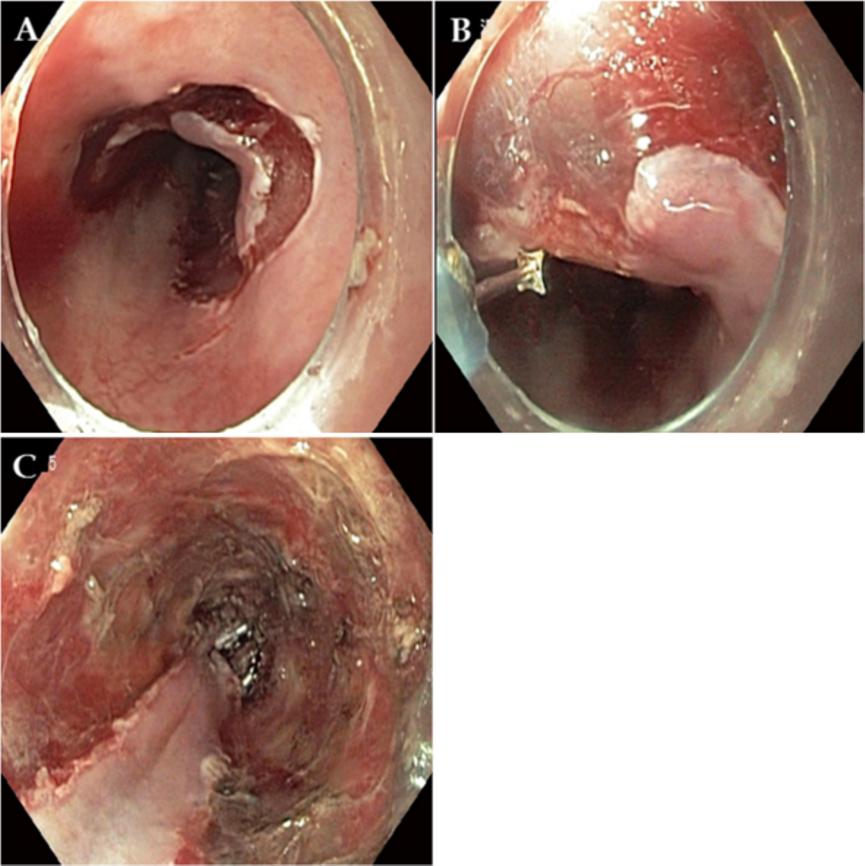
**Endoscopic submucosal dissection. (A)** Pre-cutting was performed along the outer border of the lesion. **(B)** Submucosal dissection was performed. **(C)** A large iatrogenic ulcer was observed after the procedure.

**Figure 4:**
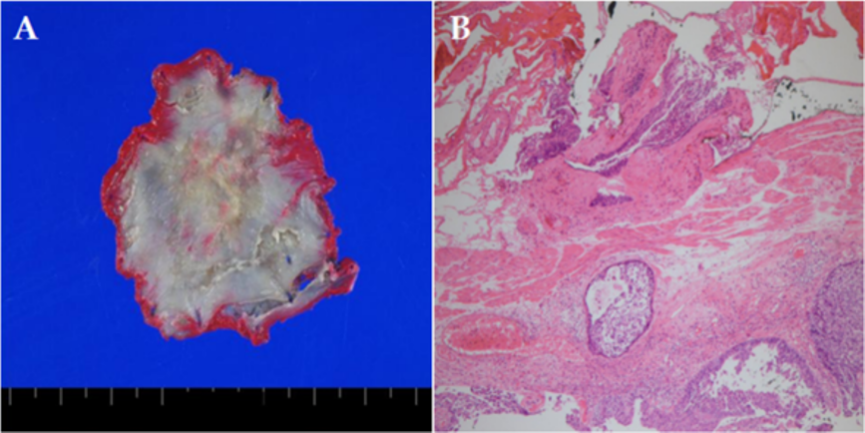
**Pathologic findings. (A)** Macroscopic finding of a specimen approximately 5cm in size after *en bloc* resection. **(B)** Squamous cell carcinoma with focal submucosal invasion.

The patient was clinically diagnosed with T1b N2 M0 G2, stage IIIA cancer (according to the American Joint Committee on Cancer (AJCC) staging system [7]) and treated with concurrent chemoradiotherapy. His chemotherapy was 5-fluorouracil plus cisplatin, and his radiotherapy of 50 Gy was delivered in 25 fractions. In a follow-up endoscopic examination performed after 5 weeks of chemoradiotherapy, we found that the lesion had decreased in size to approximately one-half the circumference of the esophageal lumen. The ulceration also demonstrated a pattern of improvement upon macroscopic observation (Figures 1C and 1D). In addition, the previously observed malignant lymph nodes were not seen on follow-up EUS, CT and PET-CT scans. After chemoradiotherapy, the patient showed a sufficient response to be clinically down-staged to T1a/T1b N0 M0 G2, stage IA (according to the AJCC staging system [7]). Though additional surgical treatment was considered with the goal of a complete cure, we acquired informed consent for ESD instead after explaining to the patient and his guardian the high post-operative risk posed by his class IV condition (American Society of Anesthesiologists classification), which was due to his poor pulmonary function (Figures 3A, 3B and 3C). A specimen measuring approximately 5×4.8cm in size was acquired through an *en bloc* resection (Figure 4A). Using the specimen, we confirmed the diagnosis of moderately differentiated squamous cell carcinoma with focal observation of submucosal invasion (sm1, shallower one-third of the submucosa) (Figure 4B).

**Figure 5:**
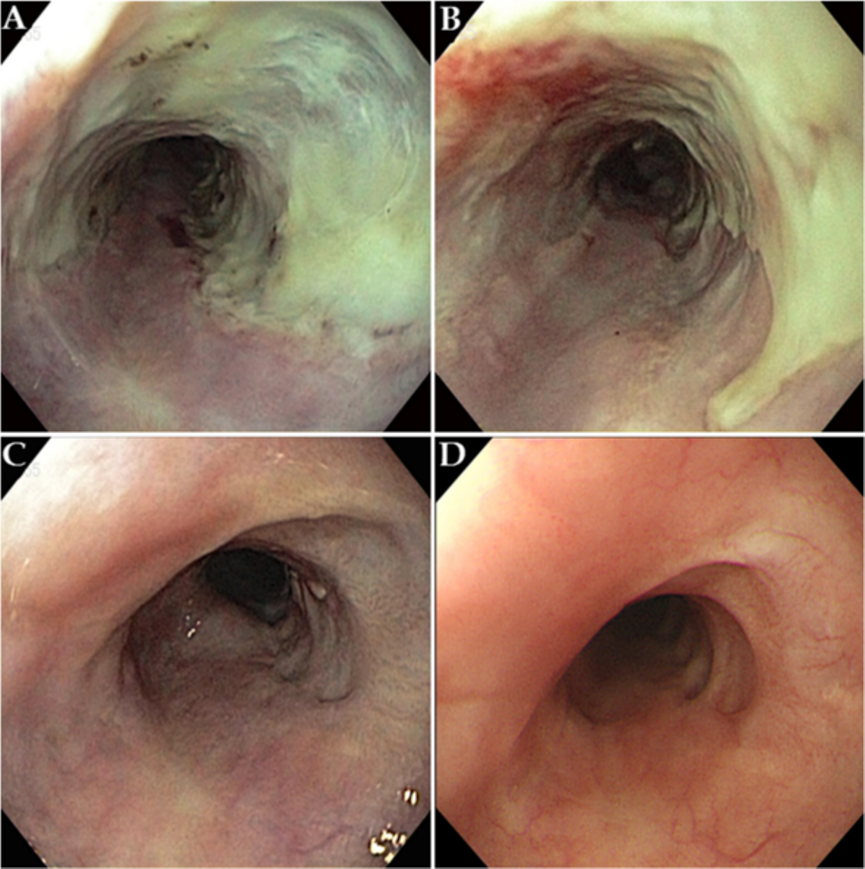
**Endoscopic findings after endoscopic triamcinolone injection. (A)** 1 week post-injection. **(B)** 1 month post-injection. **(C)** 3 months post-injection. **(D)** 6 months post-injection.

After ESD, the patient developed an iatrogenic ulcer covering nearly the total circumference of the esophageal lumen. We therefore predicted a high possibility of secondary luminal stenotic change in the future. Consequently, we administered endoscopic triamcinolone injections (ETIs) on the third, seventh and tenth days after ESD. Follow-up endoscopic examinations were performed at 1 week and at 1, 3 and 6 months after ETI (Figure 5A, 5B, 5C and 5D). They showed neither luminal stenosis nor local recurrence. In addition, esophagography and esophageal manometry tests performed 3 months after ETI did not yield findings of particular interest.

The patient has been followed on an outpatient basis and has not displayed abnormal symptoms such as dysphagia or regurgitation. Currently, neither local nor distant recurrences of esophageal cancer have been observed after 36 months.

The study protocol was approved by Daegu Fatima Hospital Ethics Committee. The patient gave us written informed consent before the procedure.

## Discussion

As is the case with the majority of gastrointestinal tract malignancies, esophageal cancer has a potentially curative therapy consisting of surgical treatment (esophagectomy with therapeutic lymphadenectomy). However, with recent reports of survival benefits [8] and complete resection rate increases in patients receiving neoadjuvant chemoradiotherapy [9], that approach has received attention as a standard treatment for locally advanced esophageal cancer [10]. Our patient had stage IIIA disease at the time of initial diagnosis, but restaging after neoadjuvant chemoradiotherapy showed a significant down-staging effect to stage IA disease.

It is well known that the surgical risk of esophagectomy varies by clinical characteristics such as age and comorbidities. Finlayson *et al*. reported that the 30-day mortality of esophagectomy was 10.7% among patients in their 60s, but that it increased to 20% among patients in their 80s [11]. Moreover, it is well known that the presence and severity of comorbidities affect disease prognosis as well as surgical outcome. Our patient was 82 years old and exhibited poor pulmonary function. Therefore, we could not perform surgical treatment, despite the fact that his esophageal cancer remained in a resectable state, and we performed ESD instead.

In actuality, endoscopic resection should be avoided in patients with esophageal cancer with submucosal invasion, because there is a substantial risk of regional lymph node metastasis. Ancona *et al*. reported confirmation of lymph node metastasis in approximately 28% of esophageal cancers with submucosal layer invasion [12]. In our patient, EUS performed during diagnosis showed invasion of the submucosal layer and three regional metastatic nodes. However, EUS performed after neoadjuvant chemoradiotherapy showed an absence of these regional lymph nodes, and ESD was planned only then. Clearly, ESD would not have been planned as a follow-up treatment if, in the initial diagnosis, there were EUS or CT findings that the lesion had invaded the muscularis propria or adventitia or any adjacent structures. Furthermore, we could not directly confirm T stage improvement by EUS prior to ESD, because radiation-induced inflammation of the esophageal wall and residual disease cannot be differentiated easily on EUS scans after neoadjuvant chemoradiotherapy [13]. However, at the time of initial diagnosis, EUS showed only submucosal invasion of the lesion. In addition, as regional lymph nodes showed improvement following neoadjuvant chemoradiotherapy, we indirectly predicted that the T staging of the lesion would not progress beyond T1b. We therefore thoroughly explained the process to the patient and his guardians and acquired informed consent to proceed with ESD.

After neoadjuvant chemoradiotherapy, the lesion area decreased significantly. However, as we performed ESD with a sufficient lateral safety margin, an iatrogenic ulcer that occupied the majority of the short axis of the esophageal lumen unavoidably developed post-operatively. We therefore performed ETI to prevent a secondary luminal stricture. The ETI was performed following a protocol described by Hashimoto *et al*. [14]. In accordance with that protocol, triamcinolone acetonide (10mg/ml) was placed in a 1-ml syringe. Then, a 25-gauge, 4mm needle (FINEMEDIX, Daegu, South Korea) was used to inject the triamcinolone in increments of 0.2ml (2mg) at 1cm intervals along the long axis and short axis of the ulcer base [14]. No luminal stricture was observed in a follow-up endoscopic examination performed after triamcinolone injection.

The patient remained in a disease-free state without local or distant recurrence 36 months after ESD. This is considered to be due to the fact that the cancer differentiation was not “poor” and the regional lymph node metastases resolved as a result of neoadjuvant chemoradiotherapy. The primary cancer site was also successfully treated to R0 resection through ESD and now shows a good prognosis.

## Conclusions

On the basis of our experience in this case, we think that ESD treatment can be considered in patients with resectable esophageal cancer if surgery poses a high risk because of comorbidities or the age of the patient and if there is a good response to neoadjuvant chemoradiotherapy. Furthermore, with respect to the large iatrogenic ulcer that occurred after ESD in our patient, we found that triamcinolone injection via endoscopy was effective in preventing luminal stricture.

## Consent

Written informed consent was obtained from the patient for publication of this case report and any accompanying images. A copy of the written consent is available for review by the Editor-in-Chief of this journal.

## References

[1] Allum WH, Stenning SP, Bancewicz J, Clark PI, Langley RE: **Long-term results of a randomized trial of surgery with or without preoperative chemotherapy in esophageal cancer.** *J Clin Oncol* 2009, **27:**5062–5067. 10.1200/JCO.2009.22.2083 10.1200/JCO.2009.22.2083 19770374

[2] Ando N, Iizuka T, Ide H, Ishida K, Shinoda M, Nishimaki T, Takiyama W, Watanabe H, Isono K, Aoyama N, Makuuchi H, Tanaka O, Yamana H, Ikeuchi S, Kabuto T, Nagai K, Shimada Y, Kinjo Y, Fukuda H, Japan Clinical Oncology Group: **Surgery plus chemotherapy compared with surgery alone for localized squamous cell carcinoma of the thoracic esophagus: a Japan Clinical Oncology Group Study—JCOG9204.** *J Clin Oncol* 2003, **21:**4592–4596. 10.1200/JCO.2003.12.095 10.1200/JCO.2003.12.095 14673047

[3] Zieren HU, Müller JM, Jacobi CA, Pichlmaier H, Müller RP, Staar S: **Adjuvant postoperative radiation therapy after curative resection of squamous cell carcinoma of the thoracic esophagus: a prospective randomized study.** *World J Surg* 1995, **19:**444–449. 10.1007/BF00299187 10.1007/BF00299187 7639004

[4] Mariette C, Piessen G, Triboulet JP: **Therapeutic strategies in oesophageal carcinoma: role of surgery and other modalities.** *Lancet Oncol* 2007, **8:**545–553. 10.1016/S1470-2045(07)70172-9 10.1016/S1470-2045(07)70172-9 17540306

[5] Sjoquist KM, Burmeister BH, Smithers BM, Zalcberg JR, Simes RJ, Barbour A, Gebski V, Australasian Gastro-Intestinal Trials Group: **Survival after neoadjuvant chemotherapy or chemoradiotherapy for resectable oesophageal carcinoma: an updated meta-analysis.** *Lancet Oncol* 2011, **12:**681–692. 10.1016/S1470-2045(11)70142-5 10.1016/S1470-2045(11)70142-5 21684205

[6] Zhang Y: **Epidemiology of esophageal cancer.** *World J Gastroenterol* 2013, **19:**5598–5606. 10.3748/wjg.v19.i34.5598 10.3748/wjg.v19.i34.5598 PMC3769895 24039351

[7] Edge S, Byrd DR, Compton CC, Fritz AG, Greene GL, Trotti A: *AJCC Cancer Staging Manual*. 7th edition. New York: Springer; 2010.

[8] Gebski V, Burmeister B, Smithers BM, Foo K, Zalcberg J, Simes J, Australasian Gastro-Intestinal Trials Group: **Survival benefits from neoadjuvant chemoradiotherapy or chemotherapy in oesophageal carcinoma: a meta-analysis.** *Lancet Oncol* 2007, **8:**226–234. 10.1016/S1470-2045(07)70039-6 10.1016/S1470-2045(07)70039-6 17329193

[9] Courrech Staal EFW, Aleman BMP, Boot H, van Velthuysen MLF, van Tinteren H, van Sandick JW: **Systematic review of the benefits and risks of neoadjuvant chemoradiation for oesophageal cancer.** *Br J Surg* 2010, **97:**1482–1496. 10.1002/bjs.7175 10.1002/bjs.7175 20645400

[10] Stahl M, Stuschke M, Lehmann N, Meyer HJ, Walz MK, Seeber S, Klump B, Budach W, Teichmann R, Schmitt M, Schmitt G, Franke C, Wilke H: **Chemoradiation with and without surgery in patients with locally advanced squamous cell carcinoma of the esophagus.** *J Clin Oncol* 2005, **23:**2310–2317. A published erratum appears in *J Clin Oncol* 2006, **24:**531 10.1200/JCO.2005.00.034 10.1200/JCO.2005.00.034 15800321

[11] Finlayson EV, Birkmeyer JD: **Operative mortality with elective surgery in older adults.** *Eff Clin Pract* 2001, **4:**172–177. A published erratum appears in *Eff Clin Pract* 2001, **4:**235 11525104

[12] Ancona E, Rampado S, Cassaro M, Battaglia G, Ruol A, Castoro C, Portale G, Cavallin F, Rugge M: **Prediction of lymph node status in superficial esophageal carcinoma.** *Ann Surg Oncol* 2008, **15:**3278–3288. 10.1245/s10434-008-0065-1 10.1245/s10434-008-0065-1 18726651

[13] Evans JA, Early DS, Chandraskhara V, Chathadi KV, Fanelli RD, Fisher DA, Foley KQ, Hwang JH, Jue TL, Pasha SF, Sharaf R, Shergill AK, Dominitz JA, Cash BD, ASGE Standards of Practice Committee: **The role of endoscopy in the assessment and treatment of esophageal cancer.** *Gastrointest Endosc* 2013, **77:**328–334. 10.1016/j.gie.2012.10.001 10.1016/j.gie.2012.10.001 23410694

[14] Hashimoto S, Kobayashi M, Takeuchi M, Sato Y, Narisawa R, Aoyagi Y: **The efficacy of endoscopic triamcinolone injection for the prevention of esophageal stricture after endoscopic submucosal dissection.** *Gastrointest Endosc* 2011, **74:**1389–1393. 10.1016/j.gie.2011.07.070 10.1016/j.gie.2011.07.070 22136782

